# Impact of Donor Obesity on Graft and Recipient Survival Outcomes After Liver Transplantation: A Systematic Review and Meta-analysis

**DOI:** 10.1097/TXD.0000000000001656

**Published:** 2024-08-29

**Authors:** Amr M.T. Alnagar, Shahab Hajibandeh, Shahin Hajibandeh, Abdul R. Hakeem, Bobby V.M. Dasari

**Affiliations:** 1 Hepatobiliary and Pancreatic Surgery and Liver Transplant Unit, Queen Elizabeth Hospital Birmingham, Birmingham, United Kingdom.; 2 Department of Hepatobiliary and Pancreatic Surgery, University Hospital of Wales, Cardiff, United Kingdom.; 3 Department of Hepatobiliary and Pancreatic Surgery, University Hospital Coventry, Coventry, United Kingdom.; 4 Department of Hepatobiliary and Liver Transplant Surgery, St James’s University Hospital NHS Trust, Leeds, United Kingdom.; 5 Institute of Immunology and Immunotherapy, University of Birmingham, Birmingham, United Kingdom.; 6 Department of Liver Transplantation, HPB Surgery, Queen Elizabeth Hospital, Birmingham, United Kingdom.

## Abstract

**Background.:**

The effect of donor body mass index (BMI) on liver transplantation (LT) outcomes remains unclear.

**Methods.:**

A systematic search of the MEDLINE, CENTRAL, Web of Science, and bibliographic reference lists was conducted. All comparative studies evaluating the outcomes of LT in obese (BMI > 30 kg/m^2^) and nonobese donors (BMI < 30 kg/m^2^) were included, and their risk of bias was assessed using the ROBINS-I assessment tool. Patient and graft survival, acute rejection, and graft failure requiring retransplantation were evaluated as outcome parameters. A random-effects model was used for outcome synthesis.

**Results.:**

We included 6 comparative studies reporting a total of 5071 liver transplant recipients from 708 obese and 4363 nonobese donors. There was no significant difference in 1-y (89.1% versus 84.0%, odds ratio [OR] 1.58; 95% CI 0.63-3.94, *P* = 0.33), 5-y (74.2%% versus 73.5%, OR 1.12; 95% CI 0.45-2.80, *P* = 0.81) graft survival, and 1-y (87.1% versus 90.3%, OR 0.71; 95% CI 0.43-1.15, *P* = 0.17) and 5-y (64.5% versus 71.6%, OR 0.71; 95% CI 0.49-1.05, *P* = 0.08) patient survival between 2 groups. Furthermore, recipients from obese and nonobese donors had a comparable risk of graft failure requiring retransplantation (OR 0.92; 95% CI 0.33-2.60, *P* = 0.88) or acute graft rejection (OR 0.70; 95% CI 0.45-1.11, *P* = 0.13).

**Conclusions.:**

A meta-analysis of the best available evidence (level 2a) demonstrates that donor obesity does not seem to have a negative impact on graft or patient outcomes. The available studies might be subject to selection bias as the grafts from obese donors are usually subject to biopsy to exclude steatosis and the recipients usually belong to the low-risk group. Future research is needed to evaluate the impact of donors subgrouped by various higher BMI on graft and patient-related outcomes as well as to capture data of the discarded grafts from obese donors; hence, selection criteria for the grafts that could be used for transplantation from obese donors is identified.

The increase in the need for liver transplantation (LT) services has created a widening disparity between the number of available liver grafts and the number of listed candidates, leading to the ineligibility of 20%–30% of the candidates for LT because of disease progression^1,2^ One of the various strategies to address graft shortage is to use extended criteria grafts, including donors with obesity.^2^

The donor body mass index (BMI) was included in the Kidney Donor Profile Index and was identified as a risk factor for unfavorable outcomes, including delayed graft function.^3^ Nevertheless, the impact of donor obesity, defined as a BMI of >30 kg/m^2^, on short-term outcomes such as reperfusion syndrome, delayed graft function, and primary nonfunction, and long-term effects such as rejection outcomes remain unclear.^4-6^ Donor BMI of >30 kg/m^2^ is the most common reason for the exclusion of potential living donors because of concerns about its strong correlation with graft steatosis.^7^

Graft steatosis, defined as the presence of macrosteatosis, is usually reported as mild, moderate, or severe if <30%, between 30% and 60%, or >60% of hepatocytes contain fat vacuoles within the cytoplasm. Macrosteatosis has been reported in 30% of deceased donor grafts, and biopsies of potential living donors have reported that 76% of donors with macrosteatosis have a BMI of >28 kg/m^2^.^8-10^ Deceased donor grafts with ≥30% macrovesicular steatosis have been linked to inferior allograft outcomes.^9^ Although BMI, a simple weight-for-height index, is commonly used to determine overweight and obesity, it is well known that the extent of graft steatosis does not necessarily correlate with BMI.^11,12^ Nevertheless, in clinical and research settings, obesity is generally defined based on BMI.

To our knowledge, there is no inclusive review and meta-analysis in the standing literature that reviews the effect of donor BMI on outcomes of LT, and only 1 previously published systematic review^13^ could be identified. Therefore, a comprehensive literature search and meta-analysis of outcomes were conducted to evaluate the impact of donor obesity on the outcomes of LT. A critical discussion of outcomes endeavored to determine the strengths and confines of available data, assess the quality of the available evidence, and identify directions for future research.

## MATERIALS AND METHODS

The eligibility criteria, methodology, and investigated outcome parameters of this study were highlighted in a review protocol and registered in PROSPERO [ID 477309]. The methodology followed the standards of the Preferred Reporting Items for Systematic Reviews and Meta-Analyses (PRISMA) statement.^14^

### Study Design

All comparative studies evaluating the outcomes of LT in obese (BMI > 30 kg/m^2^) and nonobese donors (BMI < 30 kg/m^2^) were included. Studies that did not directly compared the 2 BMI groups were excluded. Moreover, studies that did not report outcomes concerning patient BMI were excluded.

### Population of Interest

Patients of any age or sex who underwent deceased donor LT for any indication were considered for inclusion.

### Intervention of Interest

LT from an obese donor, defined as a BMI of >30 kg/m^2^, was the intervention of interest in this study. Donor obesity of any BMI group greater than, but not necessarily including, 30 kg/m^2^ was considered for inclusion if the BMI group was directly compared with a BMI group less than, but not necessarily including, 30 kg/m^2^. If a study directly compared the outcomes of >1 BMI group >30 kg/m^2^ with those of ≥1BMI groups <30 kg/m^2^, where possible, the data were pooled together based on a cutoff value of 30 kg/m^2^.

### Comparison of Interest

LT from a nonobese donor, defined as a BMI of <30 kg/m^2^ was the comparison of interest. Any donor BMI group less than, but not necessarily including, 30 kg/m^2^ was considered for inclusion if the BMI group was directly compared with a BMI group more than, but not necessarily including, 30 kg/m^2^. If a study directly compared the outcomes of >1 BMI group <30 kg/m^2^ with those of ≥1 BMI groups >30 kg/m^2^, where possible, the data were pooled together based on a cutoff value of 30 kg/m^2.^

### Outcomes

- One- and 5-y graft survival were dichotomous outcome parameters to report the proportion of grafts that survived at the end of the 1- and 5-y follow-ups.
- One- and 5-y patient survival as dichotomous outcome parameters to report the proportion of patients who survived at the end of the 1- and 5-y follow-ups.
- Graft failure requiring retransplantation.
- Acute graft rejection.

### Literature Search Strategy

A comprehensive search strategy (**Appendix 1, SDC**, http://links.lww.com/TXD/A691) was developed based on thesaurus headings, search operators, and limits in MEDLINE, CENTRAL, and the Web of Science. Two authors performed a literature search using the aforementioned electronic sources and evaluated the World Health Organization International Clinical Trials Registry (http://apps.who.int/trialsearch/), ClinicalTrials.gov (http://clinicaltrials.gov/), and ISRCTN Register (http://www.isrctn.com/) to search for ongoing and unpublished studies. Furthermore, the reference lists of the eligible studies were screened to identify potentially eligible studies. The last literature search was conducted on June 15, 2023.

### Selection of Studies

The titles and abstracts of articles found as a result of the literature search were assessed by 2 authors. When deemed necessary, the full texts of the relevant articles were retrieved and carefully assessed against the eligibility criteria of this review. Studies that met the inclusion criteria were included in the present review. Disagreements in this process were resolved through discussion between the authors. However, if disagreement persisted, an independent author was consulted.

### Data Extraction and Management

An electronic data extraction spreadsheet was created, pilot-tested in randomly selected articles, and adjusted accordingly. Two independent reviewers extracted the following information from each of the included studies.

- Study-related data.
- Baseline demographic and clinical information of the study populations.
- Primary outcomes are defined as 1- and 5-y recipient and graft survival.
- Secondary outcomes are defined as graft failure requiring retransplantation and acute graft rejection.

Discrepancies in this stage were resolved after consultation with an additional author.

### Assessment of Risk of Bias

As all the included studies were observational, assessment of their methodological quality and risk of bias were carried out by 2 authors using the Risk Of Bias in Nonrandomized Studies-of Interventions (ROBINS-I) assessment tool,^15^ which evaluates the methodological quality of observational studies in terms of the following domains: bias because of confounding, bias in the selection of participants into the study, bias in classification of interventions, bias because of deviations from intended intervention, bias because of missing data, bias in the measurement of outcomes, and bias in the selection of the reported result. Disagreements at this stage were resolved through discussion between the authors. A third reviewer was consulted if discrepancies remained unresolved.

### Summary Measures and Synthesis

For dichotomous outcome variables (1- and 5-y patient or graft survival, acute graft rejection, and graft failure requiring retransplantation), the odds ratio (OR) was determined as the summary measure. The OR is the odds of a survival event in the obese donor group compared with that in the nonobese donor group. Considering that survival is a nonadverse outcome, an OR of >1 would favor the obese donor group.

The unit of analysis for all the evaluated outcomes was an individual participant. Where possible, data on dropouts, withdrawals, and other missing information were recorded.

One reviewer independently entered the extracted data into Review Manager 5.4 software for data synthesis.^16^ A second independent author subsequently reviewed the data. Random-effects modeling was used for the analysis. The results of the analysis for each outcome parameter were reported in a forest plot with 95% confidence intervals.

Heterogeneity among studies was assessed using Cochran’s *Q* test (χ^2^). We quantified inconsistency by calculating *I*^2^ and interpreted it using the following guide: 0%–25% might not be important, 25%–75% may represent moderate heterogeneity, and 75%–100% may represent considerable heterogeneity. Moreover, when >10 studies were available in the analysis of an outcome parameter, funnel plots were constructed to assess their symmetry to visually evaluate publication bias.

Where possible, subgroup analyses were conducted for the various BMI categories. Sensitivity analyses were conducted to explore the potential sources of heterogeneity and assess the robustness of the results. Finally, the effect of each study on the overall effect size and heterogeneity was evaluated by repeating the analysis after excluding 1 study at a time (leave-one-out sensitivity analysis).

## RESULTS

**FIGURE 1.**
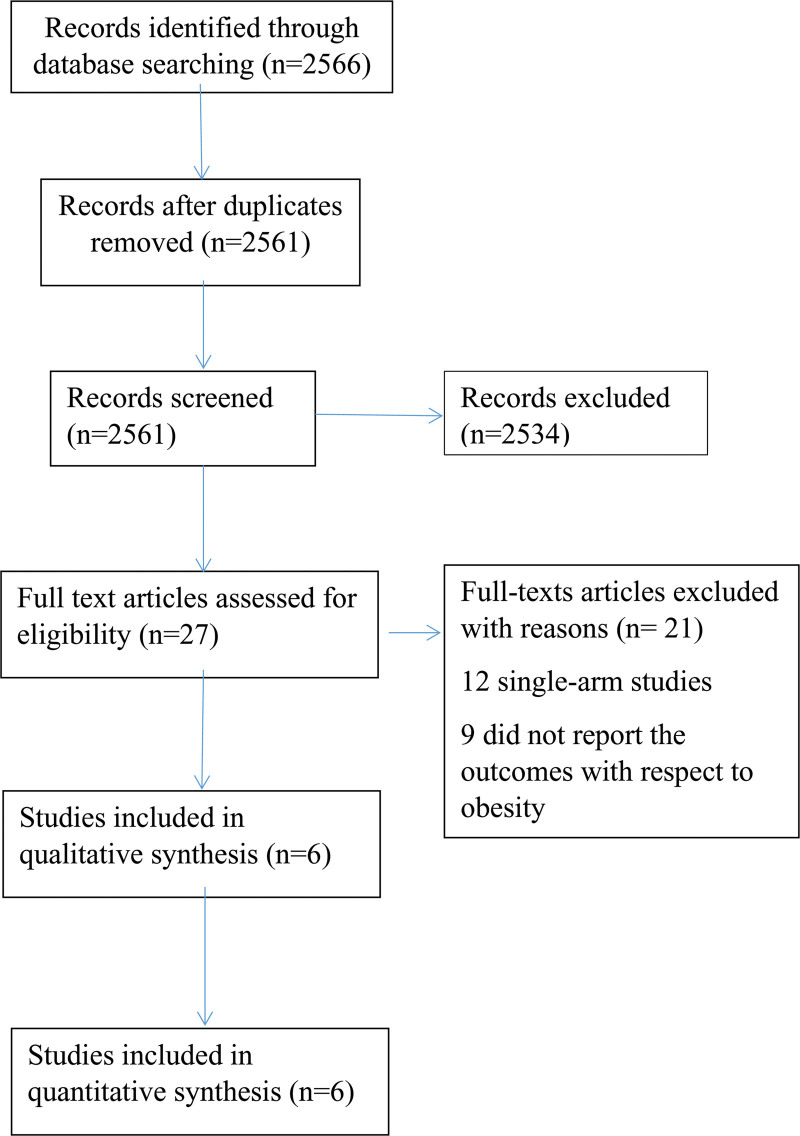
Study flow diagram.

A literature search resulted in 2566 articles. Of these, 27 studies were shortlisted for potential inclusion following the assessment of their titles, abstracts, or full texts. A further 21 studies were excluded because they were either single-arm studies or did not report donor BMI outcomes. Therefore, 6 comparative observational studies^1,17-21^ were deemed appropriate for inclusion (Figure 1). The included 6 studies reporting a total of 5071 liver transplant recipients from 708 obese and 4363 nonobese donors.

Table 1 presents the date of publication, country of origin, journal, study design, and sample sizes of the included studies. The baseline characteristics of the included patients are presented in Table 2. Generally, the included studies heterogeneously and inadequately reported baseline characteristics of the included populations.

**TABLE 1.** Included studies related data

Authors	Year	Country	Journal	Study design	Number of patients BMI >30 kg/m^2^	Number of patients BMI <30 kg/m^2^
Molina Raya et al^21^	2019	Spain	*Transplant Proc*	Retrospective observational study	50	175
Knaak et al^1^	2017	Canada	*Am J Transplant*	Retrospective observational study	105	364
Andert et al^20^	2016	Germany	*Ann Transplant*	Retrospective observational study	46	111
Dimou et al^19^	2016	US	*Surgery*	Retrospective observational study	97	2200
Bloom et al^18^	2015	US	*J Am Coll Surg*	Prospective observational study	86	292
Perito et al^17^	2012	US	*Liver Transpl*	Retrospective observational study	324	1221

BMI, body mass index.

**TABLE 2.** Baseline characteristics of the included populations

Authors	Age^*a*^ (y)	Male sex^*a*^	Donor age^*a*^ (y)	Donor BMI^*a*^	DBD^*a*^	DCD^*a*^	MELD at transplant^*a*^	CIT^*a*^ (min)	WIT^*a*^ (min)
Molina Raya et al^21^	53.32 ± 7.48 vs 53.61 ± 9.22	98.0% vs 79.4%	63.3 ± 2.26 vs 57.3 ± 1.27	32.52 ± 2.25 vs 26.06 ± 2.80	NR	NR	18.22 ± 5.25 vs 18.33 ± 5.45	NR	NR
Knaak et al^1^	54 (±11) vs 52 (±11)	64% vs 42%	37 (±11) vs 37 (±12)	33 (±2) vs 24 (±3)	NR	NR	17(±7) VS 17 (±8)	79 (±36) vs 99 (±66)	50 (±17) vs 51 (±18)
Andert et al^20^	55 (36–72) vs 55 (21–71)	61% vs 68%	59 (22–77) vs 57 (12–86)	32 (30–39) vs 26 (14–29)	NR	NR	17 (6–40) vs 16 (6–40)	465 (280–780) vs 462 (187–994)	42 (20–56) vs 44 (20–78)
Dimou et al^19^	NR	NR	NR	NR	NR	NR	NR	NR	NR
Bloom et al^18^	NR	NR	NR	NR	NR	NR	NR	NR	NR
Perito et al^17^	NR	NR	NR	NR	NR	NR	NR	NR	NR

BMI, body mass index; CIT, cold ischemia time; DBD, donation after brainstem death; DCD, donation after circulatory death; MELD, model for end-stage liver disease; NR, not reported; WIT, warm ischemia time.

### Methodological Appraisal

**FIGURE 2.**
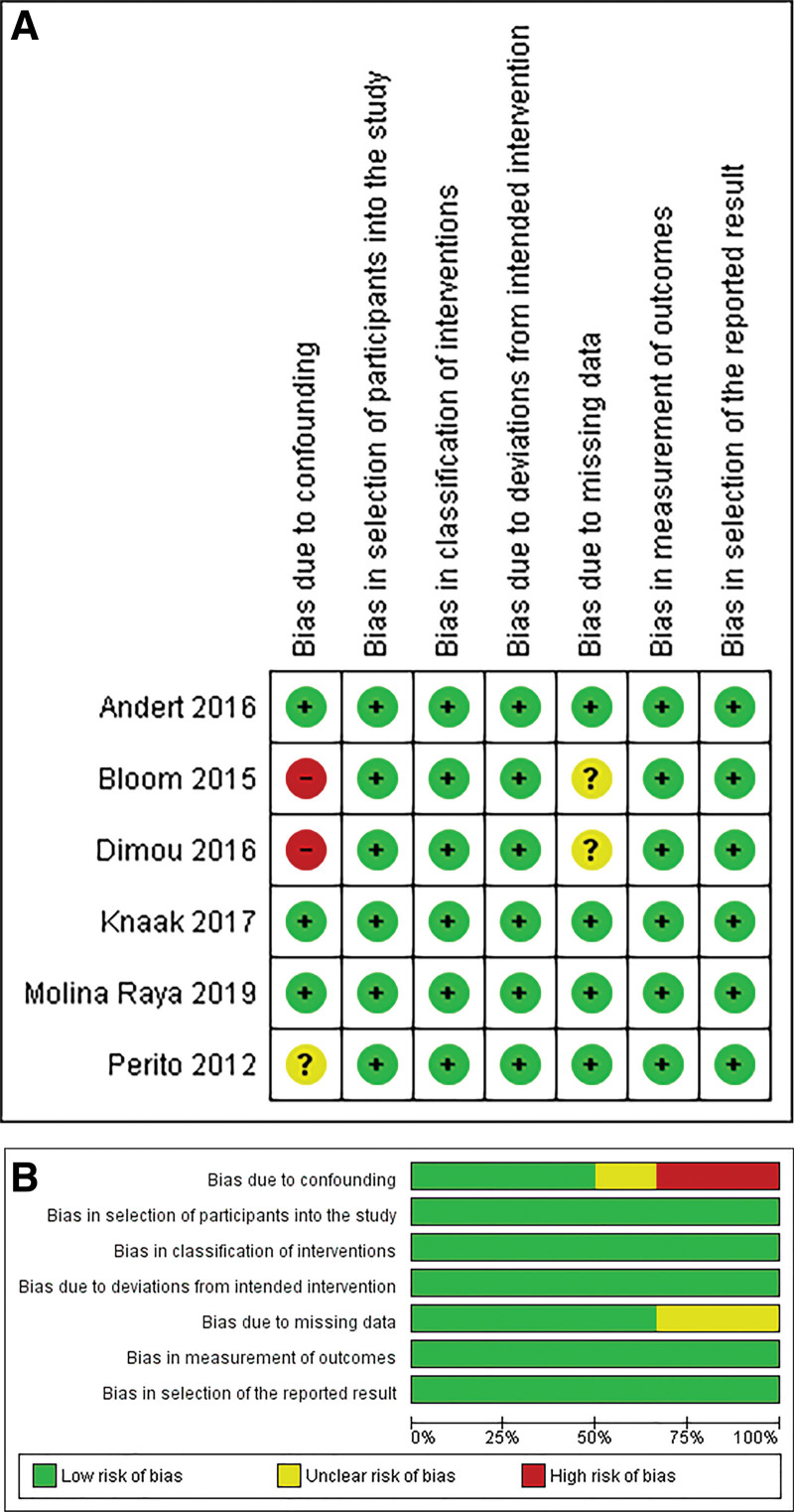
Risk of bias summary (A) and graph (B) showing authors’ judgments about each risk of bias item.

Figure 2 presents the risk of bias assessment for the included observational studies. The risk of bias because of confounding was low in 3 studies, high in 2 studies, and unclear in 1 study. The risk of bias in the selection of participants was low in all studies. Moreover, the risk of bias because of missing data was low in 4 studies and unclear in 2. The risk of bias in the measurement of outcomes was low in all studies. The risk of bias because of the classification of interventions was low in all studies. The risk of bias because of deviations from the intended intervention was low in all the studies. Finally, the risk of bias because of the selection of reported results was low in all studies.

### Outcome Synthesis

**FIGURE 3.**
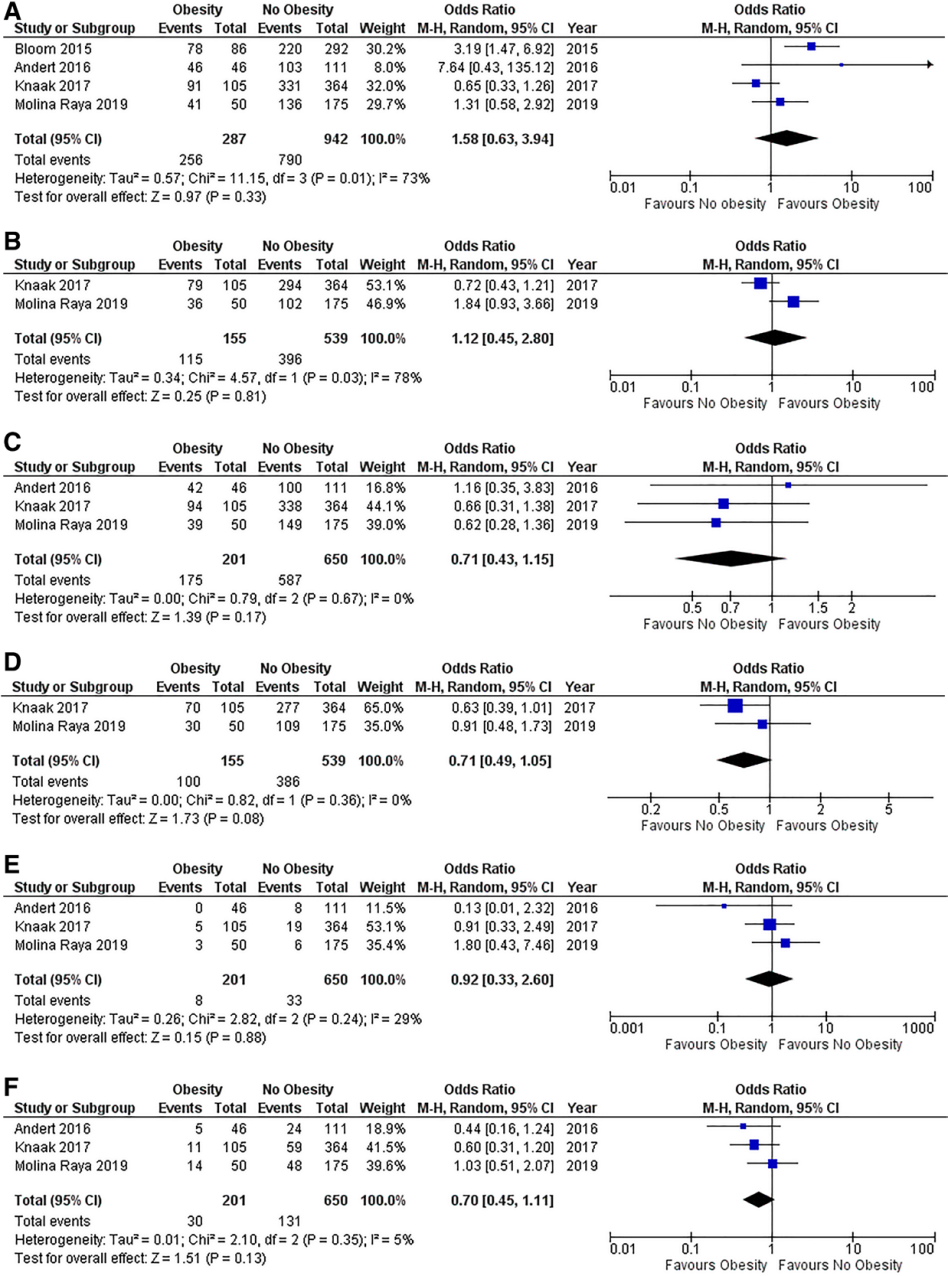
Forest plot for comparison of outcomes. Forest plots of comparison of (A) 1-y graft survival, (B) 5-y graft survival, (C) 1-y patient survival, (D) 5-y patient survival, (E) graft failure requiring retransplantation, and (F) acute graft rejection. The solid squares denote the odds ratios (ORs). The horizontal lines represent the 95% confidence intervals (CIs), and the diamond denotes the pooled effect size. M–H, Mantel–Haenszel test.

Outcomes are summarized in Figure 3.

#### Graft-related Outcome Parameters

##### One-year Graft Survival

Four studies (1229 patients) reported 1-y graft survival in the included patients. The 1-y graft survival rates were 89.1% and 84.0% in the obese and nonobese groups, respectively. There was no significant difference in 1-y graft survival between 2 groups (OR 1.58; 95% CI 0.63-3.94, *P* = 0.33). Moderate heterogeneity was observed among the evaluated studies (*I*^2^ = 73%, *P* = 0.01).

##### Five-year Graft Survival

Two studies (694 patients) reported the 5-y graft survival of their included patients. The 5-y graft survival rates were 74.2% and 73.5% in the obese and nonobese groups, respectively. There was no significant difference in the 5-y graft survival between the 2 groups (OR 1.12; 95% CI 0.45-2.80, *P* = 0.81). Moderate heterogeneity was observed among the evaluated studies (*I*^2^ = 78%, *P* = 0.03).

##### One-year Patient Survival

Three studies (851 patients) reported 1-y patient survival. The 1-y patient survival rates in the obese and nonobese groups were 87.1% and 90.3%, respectively. There was no significant difference in 1-y patient survival between the 2 groups (OR 0.71; 95% CI 0.43-1.15, *P* = 0.17). There was low heterogeneity among the evaluated studies (*I*^2^ = 0%, *P* = 0.67).

##### Five-year Patient Survival

Two studies (694 patients) reported 5-y patient survival rates. The 5-y patient survival rates were 64.5% and 71.6 % in the obese and nonobese groups, respectively. There was no significant difference in the 5-y patient survival between the 2 groups (OR 0.71; 95% CI 0.49-1.05, *P* = 0.08). There was low heterogeneity among the evaluated studies (*I*^2^ = 0%, *P* = 0.36).

##### Graft Failure Requiring Retransplantation

Three studies (851 patients) were included in the analysis of graft failure requiring retransplantation. The retransplantation rate because of graft failure was 4.0% in the obese group and 5.1% in the nonobese group. There was no significant difference in retransplantation rates between the 2 groups (OR 0.92; 95% CI 0.33-2.60, *P* = 0.88). A low degree of heterogeneity was observed (*I*^2^ = 0%, *P* = 0.45).

##### Acute Graft Rejection

Three studies (851 patients) were included in the acute graft rejection analysis. Acute graft rejection rates were 14.9% and 20.1% in the obese and nonobese groups, respectively. No significant difference in the acute graft rejection rate was found between the 2 groups (OR 0.70; 95% CI 0.45-1.11, *P* = 0.13). A low degree of between-study heterogeneity was observed (*I*^2^ = 5%, *P* = 0.35).

#### Subgroup Analysis: Comparision of Outcomes Based on Donor BMI

##### Donor BMI > 35 kg/m^2^ versus BMI < 30 kg/m^2^

Subgroup analysis of studies that reported donor BMI >35 kg/m^2^ demonstrated no significant difference in 1-y graft survival (OR 1.51, *P* = 0.45), 1-y patient survival (OR 032, *P* = 0.07), or graft failure requiring retransplantation (OR 0.96, *P* = 0.81) between the 2 groups.

### Sensitivity Analysis

The direction of the pooled effect size remained unchanged when the risk ratio or risk difference was calculated or during the leave-one-out sensitivity analysis.

## DISCUSSION

Controversy surrounds the impact of donor obesity on LT outcomes. This creates a challenging situation for transplant surgeons, particularly when a liver graft is available from an obese donor, and the recipient is in a critical clinical situation. To address this issue, we conducted a meta-analysis to assess the impact of donor obesity, defined as a BMI of >30 kg/m^2^, on LT outcomes. There was no significant difference in 1- and 5-y graft or patient survival between the 2 groups. Furthermore, recipients of obese and nonobese donors had comparable risks of acute rejection or graft failure requiring retransplantation.

The dilemma of using grafts from donors with a high BMI has recently become more frequent and is expected to grow further with the rising BMI of the donor population.^22^ According to the NHS blood and transplant activity report for 2021/2022 in the United Kingdom, 65% of donors had a BMI between 20 and 29 kg/m^2^, whereas approximately 30% of donors had a BMI >30 kg/m^2^. Despite the introduction of extended criteria donors, there have been uncertainties about utilizing liver grafts from obese donors, considering concerns of early allograft dysfunction or primary nonfunction requiring retransplantation.^23-25^ The findings from this review demonstrate that donor obesity does not increase the risk of graft failure or rejection, thereby suggesting the safety of utilizing grafts from donors with higher BMI. These results can be partly explained by the tendency of transplant centers to use grafts from obese donors for relatively lower-risk recipients, and the suggestion that the hazardous effect of high donor BMI on the liver graft is gradually built over the years because of fatty liver disease or hepatic insulin resistance,^22^ which can also explain the poorer 5-y survival among the recipients of grafts from obese (64.5%) versus nonobese donors (71.6%) although the difference is not statistically significant (*P* = 0.08). Moreover, grafts from obese donors are often subjected to liver biopsy before implantation, which typically selects grafts with acceptable steatosis grades. Furthermore, it is possible that the increased liver size in high BMI donors may compensate for the influence of moderate steatosis under ideal recipient circumstances.^22^ Therefore, these marginal grafts can help in expanding the donor pool.

It is important to differentiate between donor BMI and graft steatosis because the latter is considered a better predictor of graft function.^26^ The assessment of graft steatosis can be subjective, and standardization of reporting graft histology analysis could be associated with operator performance.^27^

Using donor BMI as an indicator of graft steatosis can potentially overcome the aforementioned limitations associated with histological tissue examination subject to addressing the controversies regarding its correlation with graft steatosis. Such potential disparity might have contributed to the findings of the study by Andert et al.^20^ in which grafts from donors with BMI 30–39 kg/m^2^ significantly increased early allograft dysfunction when compared with grafts from donors with BMI >40 kg/m^2^ and those with BMI <30 kg/m^2^. Interestingly, recipients of grafts from donors with BMI <30kg/m^2^ in the same study had a significantly higher rate of primary nonfunction requiring retransplantation when compared with patients with BMI >30 kg/m^2^. This suggests that donor BMI should be interpreted in association with other risk factors for hepatic steatosis, such as diabetes and increased donor age.^28^ The best available evidence suggests that donor obesity should not be on its own contraindication for donation.

It is imperative to exercise due caution while considering the use of these donors, taking into account the inherent biases involved. A significant number of obese donors are probably excluded because of steatosis or other extended criteria. In their study of superobese donors, Vargas et al^29^ showed that liver biopsy was conducted in 77.8% of grafts from donors with BMI ≥50 kg/m^2^, whereas only in 38.8% of the grafts in the donor BMI <50 kg/m^2^ group (*P* = 0.007). It was also noted that grafts from donors with BMI >50 kg/m^2^ are used significantly higher in recipients with hepatocellular carcinoma diagnosis who are usually less sick at the time of transplantation. As such the current literature and this review would have an inherent selection bias of using livers from only selected obese donors and not from all the offered grafts. Future studies are required where discarded grafts from obese donors are investigated for viability assessment and identify the grafts; hence, selection criteria for the grafts that could be used for transplantation.

Promising outcomes associated with the recent advancements in regional perfusion strategy and machine perfusion to optimize the function of liver grafts might potentially be options to increase the utilization of grafts from high BMI donors.^30-33^ It is well reported that static cold storage of steatotic livers is a problem, rather than the steatotic livers themselves. When a steatotic liver is preserved in static cold storage and then reperfused, there is a higher ischemia–reperfusion injury (IRI) with all its deleterious effects. The effects of IRI are worse when the cold ischemia time is increased; hence, the reason for the higher decline in these livers. On the other hand, when steatotic livers are perfused under hypothermic or normothermic conditions, the degree of IRI is lower, and hence, there is less evidence of lipolysis and sinusoidal obstruction that occurs because of the fat droplets (lipopeliosis).

The limitations of this study should be considered when interpreting these findings. Most of the included studies had a retrospective design, with an associated risk of bias. Although the main aim of a meta-analysis is to escalate the level of evidence to comprehensively evaluate the best available evidence in an effort to offer conclusions, its other mission is to demonstrate the limitations of the best available evidence so that the future better-quality studies can be designed to address such limitations. We believe our meta-analysis delivers the latter message clearly and highlights that the impact of donor obesity on survival outcomes of LT deserves high-quality research.

Although the sample size of the included studies was large, the number of included studies was relatively small. The survival outcomes were analyzed in a dichotomous fashion rather than time-to-event, as the data reported by the included studies did not allow us to analyze them in a time-to-event manner. Some studies have classified obese and nonobese donors into smaller or larger BMI intervals and have studied them separately. Although for the dichotomous outcome measures, we were able to pool the data together, for the continuous outcome measures, it was not possible to pool the data from different BMI classifications because of the high risk of bias. Furthermore, the baseline characteristics of the included populations were poorly reported in most studies. Finally, some studies used pretransplant liver biopsies more frequently in obese donors and excluded those with higher steatosis rates, which may be subject to selection bias. It should also be noted that obese donors who were offered but not used are not captured in the included studies, which hinders the refinement of selection criteria. The meta-analysis was also unable to comment on the usefulness of interventions, such as machine perfusion, which are commonly used to increase the utilization of steatotic livers.

The study conducted by Takagi et al^13^ is a systematic review that investigates the impact of donor obesity on recipients without any outcome synthesis. As a result, it cannot be compared with our study. Unlike the study by Takagi et al, we thoroughly selected the included studies based on eligibility criteria, extracted data for the purpose of outcome synthesis, and synthesized the outcome data. It is worth noting that the study by Takagi et al included several studies that do not meet the criteria to be included in a pooled analysis, and the conclusions were made without objective evaluation.

## CONCLUSIONS

A meta-analysis of the best available evidence demonstrated that donor obesity does not seem to have a negative impact on graft- or patient-related complications or survival after LT. Based on the outcomes of this study, grafts from donors with a BMI >30 kg/m^2^ can be transplanted safely, in selected recipients. However, the available studies are subject to selection bias as the grafts from obese donors are usually subject to biopsy to exclude steatosis and the recipients usually belong to the low-risk group. Future high-quality research is required to evaluate the cumulative effect of donor BMI along with other donor risk factors on recipient survival outcomes after LT and to report the degree of steatosis in the discarded grafts from obese donors. The findings of the current meta-analysis can be used to determine the sample size of future studies to provide stronger evidence in favor or against use of grafts from obese donors.

## Supplementary Material


