# Supplementary material for: Impact of Donor Obesity on Graft and Recipient Survival Outcomes After Liver Transplantation: A Systematic Review and Meta-analysis
Source: Transplant Direct. 2024 Aug 29;10(9):e1656. doi: 10.1097/TXD.0000000000001656 (PMC11365672; doi:10.1097/TXD.0000000000001656)
Supplement: Supplementary file 1 [file txd-10-e1656-s001.docx]

**Appendix 1: Search strategy.**

(("obesity"[All Fields]) OR (obese)) OR ("body mass index"[All Fields])) OR ("BMI"[All Fields])) AND (("liver transplant"[All Fields]) OR ("liver transplantation"[All Fields]))
